# Factors influencing the continuance intention of the women’s health WeChat public account: an integrated model of UTAUT2 and HBM

**DOI:** 10.3389/fpubh.2024.1348673

**Published:** 2024-06-20

**Authors:** Hewei Min, Jiaheng Li, Menglei Di, Shuhong Huang, Xinying Sun, Tao Li, Yibo Wu

**Affiliations:** ^1^Department of Social Medicine and Health Education, School of Public Health, Peking University, Beijing, China; ^2^School of Basic Medical Sciences, Hebei University, Baoding, Hebei, China; ^3^Department of Gynaecology and Obstetrics, The Fourth Central Hospital of Baoding City, Baoding, Hebei, China; ^4^Tianjiazhuang Village Clinic, Baoding, Hebei, China

**Keywords:** health communication, women health, mobile health, continuous usage, integrated model

## Abstract

**Background:**

Women’s health WeChat public accounts play a crucial role in enhancing health literacy and fostering the development of healthy behaviors among women by disseminating women’s health knowledge. Improving users’ continuous usage behavior and retention rates for the women’s health WeChat public account is vital for influencing the overall effectiveness of health communication on WeChat.

**Objective:**

This study aimed to construct a comprehensive model, delving into the key factors influencing women’s continuance intention of the women’s health public accounts from the perspectives of perceived health threats, individual abilities, and technological perceptions. The goal is to provide valuable insights for enhancing user stickiness and the effectiveness of health communication on WeChat public accounts.

**Method:**

An online survey was conducted among women receiving gynecological care at a certain hospital to gage their willingness for sustained use of the women’s health WeChat public accounts. Through structural equation modeling, the study investigated the influencing factors on women’s sustained intention to use the women’s health WeChat public accounts.

**Results:**

The study included a total of 853 adult women. Among them, 241 (28.3%) women had followed women’s health official accounts in the past but do not currently follow them, 240 (28.1%) women had followed women’s health official accounts in the past and are still following them, and 372 (43.6%) women had never followed women’s health official accounts. Currently, 240 women are still browsing women’s health public accounts, 52 of whom read women’s health public accounts every day, and most of them read women’s health public accounts for 10–20 min at a time (100, 11.7%). The results of the structural equation model revealed that performance expectancy, social influence, hedonic motivation, habit, and e-health literacy had significantly positive effects on women’s sustained intention to use public accounts (performance expectancy: *β* = 0.341, *p* < 0.001; social influence: *β* = 0.087, *p* = 0.047; hedonic motivation: *β* = 0.119, *p* = 0.048; habit: *β* = 0.102, *p* < 0.001; e-health literacy: *β* = 0.158, *p* < 0.001). E-health literacy and self-efficacy indirectly influence sustained intention by affecting performance expectancy, effort expectancy, social influence, facilitating conditions, hedonic motivation, and habit. The effect sizes of e-health literacy on performance expectancy, effort expectancy, social influence, facilitating conditions, hedonic motivation, and habit were 0.244 (*p* < 0.001), 0.316 (*p* < 0.001), 0.188 (*p* < 0.001), 0.226(*p* < 0.001), 0.154 (*p* < 0.001), and 0.073 (*p* = 0.046). The effect sizes of self-efficacy on performance expectancy, effort expectancy, social influence, facilitating conditions, hedonic motivation, and habit were 0.502 (*p* < 0.001), 0.559 (*p* < 0.001), 0.454 (*p* < 0.001), 0.662 (*p* < 0.001), 0.707 (*p* < 0.001), and 0.682 (*p* < 0.001). Additionally, perceived severity and perceived susceptibility indirectly affected sustained intention by influencing performance expectancy and social influence. The effect sizes of perceived severity on performance expectancy and social influence were 0.223 (*p* < 0.001) and 0.146 (*p* < 0.001). The effect size of perceived susceptibility to social influence was 0.069 (*p* = 0.042).

**Conclusion:**

Users’ e-health literacy, self-efficacy, perception of disease threat, and users’ technological perceptions of the WeChat public accounts are critical factors influencing women’s continuance intention of using the WeChat public accounts. Therefore, for female users, attention should be given to improving user experience and enhancing the professionalism and credibility of health information in public account design and promotion. Simultaneously, efforts should be made to strengthen users’ health awareness and cultivate e-health literacy, ultimately promoting sustained attention and usage behavior among women toward health-focused public accounts.

## 1 Introduction

### 1.1 Overview

Women’s health, encompassing reproductive health, mental wellbeing, self-care, and equal access to medical services, is a focal point of societal concern. Disseminating and popularizing knowledge about women’s health, along with the attention and maintenance of women’s physical, mental, and social wellbeing, constitute crucial issues in the field of women’s health.

With the rapid development of information technology, online social platforms play a vital role in the dissemination of health information. WeChat, a social app used by hundreds of millions of people in China, features public accounts that facilitate one-to-many information dissemination and communication through mass messaging, forwarding, sharing, and background interactions. According to the “2022 National Health Insight Report,” WeChat public accounts are the primary channel for people to obtain health knowledge, with an adoption rate of 84% (1). WeChat public accounts have become one of the most popular platforms for accessing health information in China (2). In the field of women’s health, an increasing number of doctors and medical institutions use WeChat public accounts to disseminate knowledge related to women’s health, including knowledge on the prevention and treatment of female-specific diseases (such as breast diseases and uterine diseases), women’s physiological health, pregnancy and the postpartum period, and sexual education. These WeChat public accounts are known as women’s health WeChat public accounts, such as the public accounts “Consulting room_11” and “DingXiangMaMi,” which act as a crucial channel for the dissemination and popularization of women’s health knowledge.

However, despite the abundance of health communication public accounts, this does not necessarily imply that health information is fully disseminated and utilized. WeChat public accounts, after experiencing rapid initial user growth, often face challenges in user retention, leading to phenomena such as decreased readership and a decline in sustained active users. A retrospective cohort study conducted by Helander et al. indicated that among 189,770 users who had downloaded a mobile health app, only 2.58% actively used it within the first week (3). Furthermore, as time progresses, there is a significant decline in user visits to mobile health apps (4). Nonetheless, Bhattacherjee pointed out that the long-term survival and success of information technology or information systems depend not on initial user adoption but on continuous usage (5). Forman et al. suggested that 80% of older diabetes patients express willingness to accept mobile health technology once they receive support and persist in its use (6). Therefore, understanding how to promote sustained user attention and usage of WeChat public accounts is a crucial research direction for improving the dissemination effectiveness of WeChat public accounts and enhancing public health literacy.

The willingness for sustained usage refers to the user’s intention to continue using an information system after the initial use, serving as a crucial driver for continued engagement. Research on the factors and mechanisms influencing the sustained usage intention of mobile health technology has been gradually increasing. Wang et al. included 58 cross-sectional studies, which indicated that attitude, satisfaction, health empowerment, perceived usefulness, and perceived quality of life have significant direct effects on the sustained usage intention of mobile health technology (7). Additionally, factors such as geographic region, user type, mHealth type, user age, and publication year play significant moderating roles in the relationships between influencing factors such as trust and sustained usage intention. Therefore, personalized promotional strategies need to be designed based on the factors influencing users’ sustained usage intentions to enhance user retention in mobile health technology.

Previous studies highlight that improving users’ sustained usage intentions for communication platforms is a crucial prerequisite for effective and sustainable health communication. Currently, research on the sustained usage intention of mobile health technology mainly focuses on health apps (8, 9), wearable devices (10, 11), and remote medical technologies (12, 13). However, as a vital channel for health promotion, research on health-related WeChat public accounts primarily centers on the functionalities and content of the accounts, lacking an exploration of users’ sustained usage intentions.

Hence, the purpose of this study is to understand users’ intentions for sustained usage of the women’s health WeChat public accounts. Drawing on the Technology Acceptance and Integration Model 2 and the health belief model (HBM), this study aimed to explore the factors influencing the public’s sustained usage intention for women’s health public accounts. The findings will contribute to expanding the theoretical understanding of women’s health information dissemination, optimizing the operation and information quality of health-related public accounts, increasing user engagement, and ultimately improving the effectiveness and sustainability of women’s health information dissemination, thereby raising the level of women’s health literacy.

### 1.2 Theoretical models

**Figure 1:**
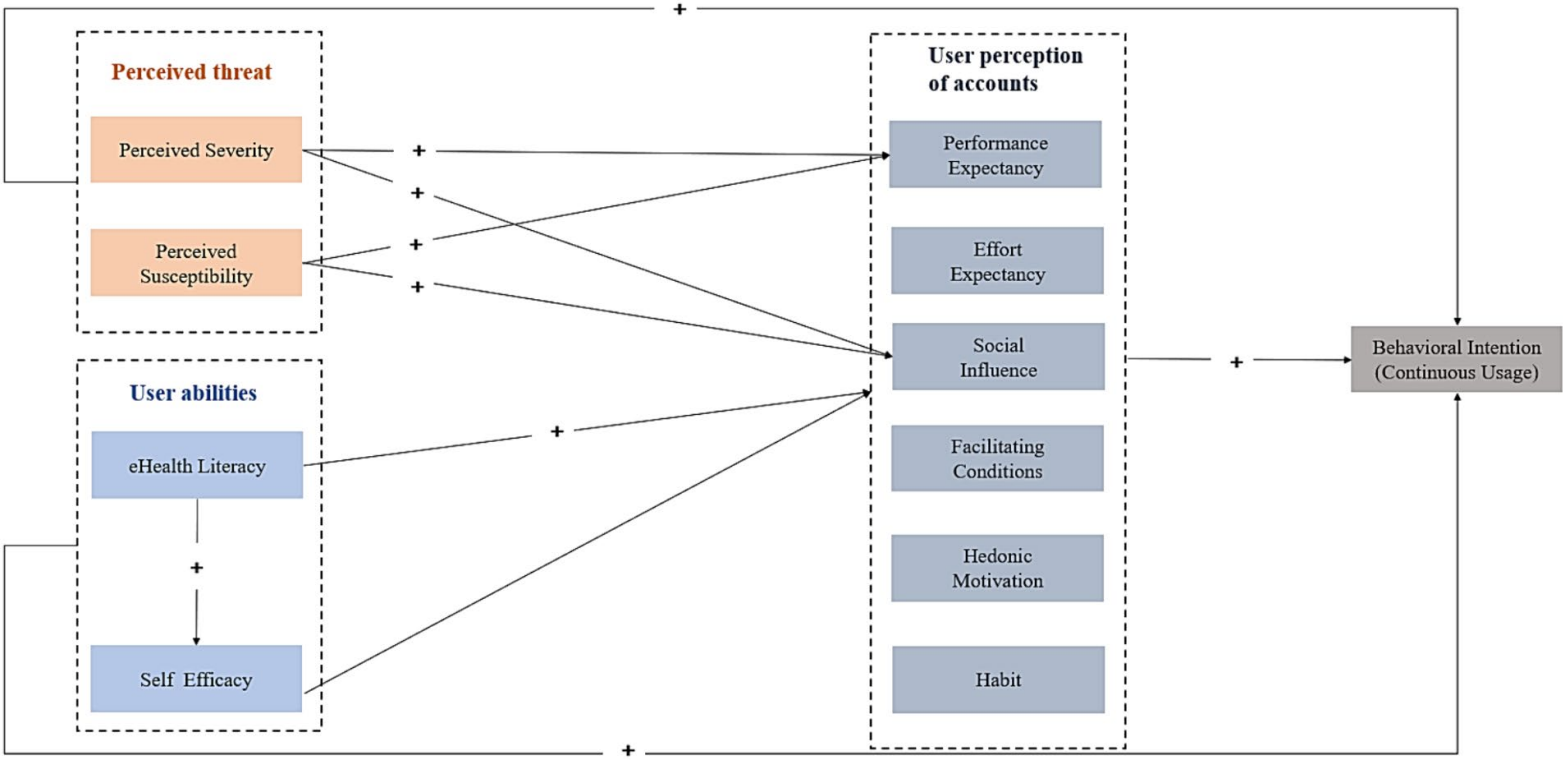
Hypostasis of the study.

This study explored factors influencing women’s sustained intention to use women’s health WeChat public accounts from the perspectives of perception of disease, perception of technology, and user abilities (self-efficacy and eHealth literacy). On the one hand, the study adopted the Unified Theory of Acceptance and Use of Technology 2 (UTAUT2) model to explain the influence of users’ continuance intention of the Women’s Health WeChat public accounts. On the other hand, considering that the public’s continued use of health-related WeChat public accounts is a health behavior, the users’ attitudes and beliefs about health knowledge, especially the assessment of the threat of a specific disease and their own coping ability, are important factors that influence the adoption and continued reception of health information. Therefore, to address the limitations of UTAUT2, this study combined the HBM and eHealth literacy to extend the UTAUT2 model from the perspectives of user abilities and disease perception. The following sections provide more details on the rationale for selecting this theoretical model, and the research hypotheses are depicted in Figure 1.

#### 1.2.1 UTAUT2

The UTAUT2 model, proposed by Venkatesh et al. in 2012 (14) as an extension of UTAUT (15), is designed to explain the intention to adopt information technology. This model categorizes factors influencing users’ behavior and intention to use information technology into seven core variables: performance expectancy, effort expectancy, social influence, facilitating conditions, hedonic motivation, perceived value, and habit. It also introduces three moderating variables: gender, age, and experience. Research indicates that the UTAUT2 model explains approximately 74% of consumer behavioral intention differences and 52% of consumer technology usage differences for the focal technology (16). In recent years, the UTAUT2 model has been increasingly applied to the study of usage behavior in mobile health technology, such as physiological indicators, mobile monitoring technology (17), and healthcare wearable technology (18). Furthermore, Lin et al., building upon UTAUT2, added perceived health threat, showing that the older adults’ sustained intentions to use maintenance-oriented WeChat official accounts are influenced by performance expectancy, hedonic motivation, social influence, and threat assessment (19). Therefore, this study utilized the UTAUT2 model to explore the technology-perceived factors of women’s health public accounts’ persistent usage intentions. Because women’s health WeChat public accounts are often accessible to the public for free, this study excluded the price value from UTAUT2.

#### 1.2.2 HBM

The HBM, initially proposed by social psychologist Rosenstock (20) and later refined by Becker and Maiman (21), is one of the earliest theoretical models used in individual health behavior prediction. This model emphasizes the use of individual attitudes and beliefs to explain health behaviors, asserting that factors influencing an individual’s adoption of specific health-related behaviors depend primarily on two aspects: threat assessment and outcome expectations. Additionally, the model suggests that cues to action and self-efficacy are crucial factors in shaping health behavior. Originally used to explain users’ preventive health behaviors, the HBM has been extended to explain and predict various long-term and short-term risky health behaviors. As people become more aware of health threats, they tend to focus on health information and adopt health behaviors, including using smartphones for chronic disease management (22) and utilizing electronic health services for disease prevention (23). Therefore, HBM has progressively been applied to the study of health information behaviors, such as consumer acceptance of online mental health resources (24) and online health information services (25). Considering that women’s perceptions of their health status and their ability to use mobile health technology (26) may influence their willingness to continue using women’s health WeChat public accounts, the hypothetical model of this study incorporated threat perception and self-efficacy from the HBM.

Past research indicated that individuals with high perceived threat tend to have higher health motivation and higher performance expectations for health technology (27). Additionally, they are also more likely to be influenced by social factors, which enhances their willingness to use health technology (28). Regarding self-efficacy, previous studies have considered it an extended factor of the UTAUT model, indicating that individuals with high self-efficacy, who are confident in their ability to use new technology, can positively influence performance expectancy, effort expectancy, social influence, and facilitating conditions (29). Furthermore, research suggests that mobile self-efficacy has a significant positive impact on the perceived enjoyment of mobile learning (30). Therefore, this study also explored the influence of perceived severity, perceived susceptibility, and self-efficacy on the UTAUT2 model.

#### 1.2.3 eHealth literacy

EHealth literacy refers to an individual’s ability to use information and communication technology to access, understand, evaluate, and apply health information, utilizing the acquired knowledge to address health-related issues (31). On the one hand, individuals with high eHealth literacy exhibit greater familiarity and proficiency in using mobile health technologies, making it a significant factor influencing the acceptance and utilization of mobile medical technologies (32). On the other hand, as a capability to utilize mobile technology for acquiring and assessing health knowledge, the impact of eHealth literacy on the intention to use mobile health technology involves individuals’ perception and evaluation of the technology itself. Past research has suggested that eHealth literacy is a positive predictor of perceived usefulness of eHealth technology (33). Users with lower eHealth literacy tend to have lower perceptions of the usefulness and ease of use of health information systems (34). Consequently, they are inclined to have lower performance expectancy, effort expectancy (35), and facilitating conditions, and are less likely to find enjoyment in the technology itself. Moreover, due to their limited ability to use mobile health technology, even in a positive social environment, their intention to use such technology may not be strong.

Furthermore, previous studies indicated that eHealth literacy significantly influences self-efficacy. Individuals with high eHealth literacy demonstrate strong capabilities in utilizing mobile technology to access and apply health information, fostering confidence in their ability to use such technology (36–38). Therefore, we hypothesized that eHealth literacy could positively influence continuance intention, self-literacy, and the UTAUT2 model.

## 2 Materials and methods

### 2.1 Research object

This is a cross-sectional study based on an online questionnaire survey conducted from December 2022 to February 2023 among women visiting the gynecology department of a hospital in Hebei Province, China. The study has received approval from the Ethics Committee of the Fourth Central Hospital of Baoding City (Approval No. 2022013), and all participants provided informed consent before participating in the survey. The inclusion criteria for the study subjects are as follows: age ≥ 18 years; female; Chinese nationality; owns a smartphone, uses WeChat, and follows WeChat official accounts; can independently or with the assistance of an investigator complete the online questionnaire survey; basic literacy skills with unimpeded communication abilities; voluntarily participates in the study and signs the informed consent form. Patients with any of the following criteria were excluded: confusion or mental abnormalities, cognitive impairment, participation in other similar research projects, and unwillingness to collaborate.

A total of 1,281 questionnaires were distributed for this survey, with 1,216 valid responses, resulting in an effective rate of 94.9%. The final analysis focused on 853 respondents who had browsed or obtained knowledge about women’s health through women’s health WeChat official accounts.

Previous research has shown that the sample size for structural equation modeling can be determined by the number of parameters within the model (39, 40). In general, a sample size of 10–20 times the number of parameters is acceptable. The minimum sample size required for this study is 220. Therefore, the number of subjects included in this study is sufficient.

### 2.2 Data collection

The first section of the questionnaire included a screening question: “Have you ever obtained health information through women’s health WeChat official accounts?.” If participants answered “no,” the questionnaire was considered invalid. The second part consisted of socio-demographic variables, including age, place of residence, highest educational level, and marital status. The third part comprised latent variable items, using mature scales from domestic and international sources, adapted to be relevant to women’s health WeChat official accounts. Expert consultations and pre-surveys were conducted to ensure the questionnaire’s reliability and validity. Specifically:

(1) UTAUT2: Adapted from the original scale by Venkatesh et al. (14) and previous studies (19, 41, 42), this section measured the public’s intention to sustain the use of women’s health WeChat official accounts. It consisted of 7 dimensions with 25 items: behavioral intention (3 items), performance expectancy (3 items), effort expectancy (4 items), social influence (4 items), facilitating conditions (3 items), hedonic motivation (3 items), and habit (4 items). All items used a 5-point Likert scoring system. Higher scores in each dimension indicated a stronger intention. Detailed explanations of each dimension are provided in Table 1, and confirmatory factor analysis results are shown in Tables 2, 3.
(2) eHealth literacy scale (eHEALS): The eHEALS was used to measure the public’s ability to search, comprehend, and contextualize health information through web-based technologies (31). The scale comprised eight items, scored on a 5-point Likert scale. The total score ranged from 0 to 50, with higher scores indicating higher eHealth literacy. This study used the Chinese version of the eHEALS scale by Guo et al. (44). Confirmatory factor analysis results are in Table 2, with a Cronbach’s α coefficient of 0.920 and item factor loadings between 0.669 and 0.875.
(3) HBM section: Based on the research hypotheses, three dimensions were selected: perceived severity, perceived susceptibility, and self-efficacy, each with 3 items, totaling 12 items. The scoring system was a 5-point Likert method, with a maximum score of 10 for each subscale. Higher scores indicated a more prominent trait in that dimension. Detailed explanations for each dimension are provided in Table 1. Cronbach’s α coefficients for perceived severity, perceived susceptibility, and self-efficacy were 0.928, 0.934, and 0.934, respectively. Item factor loadings ranged from 0.836 to 0.943.

**Table 1:** Constructs and items included in the UTAUT2 scale.

Construct	Item	Measurement	Source
Behavioral intention	BI1	I intend to continue to follow the women’s health WeChat official account in the future.	(14, 19, 41)
	BI2	I will continue to use the women’s health WeChat official account at least as often as I do now.	
	BI3	I will increase the frequency of using the women’s health WeChat official account in the future.	
Performance expectancy	PE1	Women’s health WeChat official accounts are helpful for me to understand medical knowledge	
	PE2	Women’s health WeChat official accounts are helpful to me in preventing and/or dealing with diseases	
	PE3	Women’s health WeChat official accounts help me to keep healthy	
Effort expectancy	EE1	Learning how to use women’s health WeChat official accounts is easy for me	
	EE2	Using women’s health WeChat official accounts is easy for me	
	EE3	Reading the articles on women’s health WeChat official accounts is easy for me	
	EE4	It’s easy for me to find the information I need through Women’s health WeChat official accounts.	
Social influence	SI1	I will be influenced by my partner and parents to follow women’s health WeChat official accounts.	
	SI2	I will be influenced by my friends to follow women’s health WeChat official accounts.	
	SI3	I will be influenced by colleagues and people around me to follow women’s health WeChat official accounts.	
	SI4	I will be influenced by the mass media and advertising and promotion to follow women’s health WeChat official accounts.	
Facilitating conditions	FC1	I have the knowledge to follow and use Women’s Health WeChat official accounts.	
	FC2	I can get help when I have problems using women’s health WeChat official accounts.	
	FC3	I can get help when I have problems understanding articles on women’s health WeChat official accounts.	
Hedonic motivation	HM1	Reading articles on women’s health WeChat official accounts can bring me enjoyment.	
	HM2	Reading articles on women’s health WeChat official accounts makes me feel good.	
	HM3	Reading articles on women’s health WeChat official accounts helps me to spend my free time	
Habit	HA1	Using women’s health WeChat official accounts has become a habit for me	
	HA2	I have a sense of dependence on women’s health WeChat official accounts.	
	HA3	When I need health information or health services, I think of women’s health WeChat official accounts first.	
	HA4	I feel that following women’s health WeChat official accounts has become (will become) a necessity in my life.	
Perceived severity	PSE1	If I have a gynecological disease, it would cause me distress.	
	PSE2	If I have a gynecological disease, it would cause me great concern.	
	PSE3	If I have a gynecological disease, it would capture my attention.	
Perceived susceptibility	PSU1	I am at risk of having a gynecological disease.	(43)
	PSU2	It is likely that I will have a gynecological disease.	
Self-efficacy	SE1	I am confident that in the face of challenges such as difficulties in navigation or comprehension, I can still follow and utilize the women’s health WeChat official account.	
	SE2	I have confidence in my ability to understand the information presented in the women’s health WeChat official account.	
	SE3	I am confident that I can obtain the information I seek from the women’s health WeChat official account.	
	SE4	I am confident in effectively utilizing the content and services provided by the women’s health WeChat official account.	

**Table 2:** Participants’ subscriptions to the women’s health public numbers (*N* = 853).

Subscription	Frequency	Percentage %
Subscribed in the past and not currently subscribed	241	28.3
Subscribed in the past and currently subscribed	240	28.1
Never subscribed	372	43.6

**Table 3:** Frequency of browsing the women’s health WeChat official accounts among the participants (*N* = 853).

Browsing	Frequency	Percentage %
Not browsing	613	71.9
Daily	52	6.1
5–6 days/week	39	4.6
3–4 days/week	36	4.2
1–2 days/week	66	7.7
1–3 days/month	30	3.5
Less than 1 day/month	17	2.0

### 2.3 Quality control

Prior to the formal survey, a preliminary survey was conducted. Trained researchers distributed questionnaires face-to-face to participants. Every Sunday evening, the project leader communicated with the researchers to consolidate, evaluate, and provide feedback on the collected questionnaires. After questionnaire retrieval, two individuals conducted logical checks and data screening back-to-back. If outliers were identified during data analysis, the original questionnaire needed to be located or verified with the surveyors before proceeding to the next analysis step.

### 2.4 Data analysis

Statistical analysis was performed using SPSS 26.0 (SPSS Inc., an IBM Company, Chicago, Illinois, United States) and M*plus* (version 7.4, Muthén & Muthén, Los Angeles, CA, United States) software. Categorical variables were presented using frequencies and percentages. M*plus* 7.4 was used for confirmatory factor analysis of the questionnaire and for constructing structural equation models. The significance level was set at α = 0.05, and the maximum likelihood robust (MLR) estimator (45) was used for analyzing non-normally distributed data.

## 3 Results

### 3.1 Basic information

A total of 853 adult females were included in this study. Among them, 133 individuals were aged 18–24 (15.6%), 344 were aged 25–34 (40.3%), 277 were aged 35–44 (32.5%), 83 were aged 45–54 (9.7%), and 16 were aged 55–64 (1.9%). Other demographic information is presented in Table 4.

**Table 4:** Demographic characteristics of the participants (*N* = 853).

Variable	Category	Frequency	Percentage %
Age	18–24	133	15.6
	25–34	344	40.3
	35–44	277	32.5
	45–54	83	9.7
	55–64	16	1.9
Location	Urban	562	65.9
	Rural	291	34.1
Education	Junior high school and below	222	26.0
	High school	78	9.1
	Vocational school	294	34.5
	College and above	259	30.4
Marital status	Unmarried	110	12.9
	Married	727	85.2
	Divorced	13	1.5
	Widowed	3	0.4

### 3.2 Usage of women’s health WeChat’s official accounts

The participants’ subscriptions to the women’s health WeChat official accounts are shown in Table 2. Among them, 241 (28.3%) women had followed women’s health official accounts in the past but did not currently follow them, 240 (28.1%) women had followed women’s health official accounts in the past and were still following them, and 372 (43.6%) women had never followed women’s health official accounts.

The frequency of browsing the women’s health WeChat official accounts among the participants is shown in Table 3. Among them, 613 (71.9%) females were not currently following women’s health public accounts, and therefore the frequency of reading was 0. There were 52 (6.1%) females browsing women’s health public accounts daily, 39 (4.6%) females browsing women’s health public accounts 5–6 days per week, and 36 (4.2%) females browsing women’s health public accounts 3–4 days per week. Furthermore, there were 66 (7.7%) females browsing women’s health public accounts 1–2 days per week, 30 (3.5%) women browsing women’s health public accounts 1–3 days per month, and 17 (2.0%) women reading women’s health public websites less than 1 day per month.

The length of time the participants spend browsing the women’s health WeChat official accounts each time is shown in Table 5. Among them, 613 (71.9%) females were not currently following women’s health public accounts, and therefore the length of reading was 0. Meanwhile, there were 88 (6.1%) females reading women’s health public accounts for 0–10 min each time, 100 (11.7%) females reading women’s health public accounts for 10–20 min, 40 (4.7%) females reading women’s health public accounts for 20–30 min, and 12 (1.4%) females reading women’s health public accounts for more than 30 min.

**Table 5:** Length of time the participants spent browsing the women’s health public numbers (*N* = 853).

Browsing	Frequency	Percentage %
Not browsing	613	71.9
0–10 min	88	10.3
10–20 min	100	11.7
20–30 min	40	4.7
>30 min	12	1.4

### 3.3 Reliability and validity

We measured the reliability and validity of the UTAUT2, and the results are presented in Tables 6, 7. The internal consistency, as indicated by Cronbach’s alpha values, ranged from 0.899 to 0.959, demonstrating good internal consistency for each dimension of the UTAUT2 scale. The standardized factor loadings of UTAUT2 items ranged from 0.756 to 0.957, indicating a good correspondence between factors and items. The composite reliability (CR) for each dimension ranged from 0.891 to 0.960, suggesting excellent structural reliability for the scale (46). The average variance extracted (AVE) values, ranging from 0.705 to 0.889, indicated good convergent validity for the scale (47). Additionally, the correlations between dimensions of the UTAUT2 scale were lower than the square roots of AVE, demonstrating good discriminant validity for the scale (48). The measurement model for constructing the UTAUT2 scale showed good fit indices: χ^2^ = 888.136, df = 231, χ^2^/df = 3.845, CFI = 0.968, TLI = 0.962, and RMSEA = 0.058.

**Table 6:** Confirmatory factor analysis results for the UTAUT2 scale.

Construct	Items	Item loading	Cronbach’s alpha	Composite reliability (CR)	Average variance extracted (AVE)
Behavioral intention	BI1	0.890	0.916	0.891	0.731
	BI2	0.823			
	BI3	0.850			
Performance expectancy	PE1	0.928	0.959	0.960	0.889
	PE2	0.957			
	PE3	0.943			
Effort expectancy	EE1	0.887	0.935	0.936	0.784
	EE2	0.912			
	EE3	0.884			
	EE4	0.858			
Social influence	SI1	0.776	0.899	0.905	0.705
	SI2	0.911			
	SI3	0.907			
	SI4	0.751			
Facilitating conditions	FC1	0.811	0.917	0.923	0.801
	FC2	0.930			
	FC3	0.938			
Hedonic motivation	HM1	0.919	0.908	0.911	0.775
	HM2	0.930			
	HM3	0.785			
Habit	HA1	0.899	0.926	0.927	0.761
	HA2	0.882			
	HA3	0.824			
	HA4	0.883			

**Table 7:** Correlation matrix and square root of AVE of the UTAUT2 scale.

	BI	PE	EE	SI	FC	HM	HA
BI	0.855						
PE	0.684	0.943					
EE	0.598	0.679	0.885				
SI	0.568	0.612	0.595	0.840			
FC	0.649	0.716	0.688	0.700	0.895		
HM	0.618	0.615	0.668	0.603	0.716	0.880	
HA	0.550	0.492	0.535	0.583	0.665	0.743	0.872

BI, Behavioral intention; PE, performance expectancy; EE, effort expectancy; SI, social influence; FC, facilitating conditions; HM, hedonic motivation; HA, habit.

### 3.4 Structural equation model

**Figure 2:**
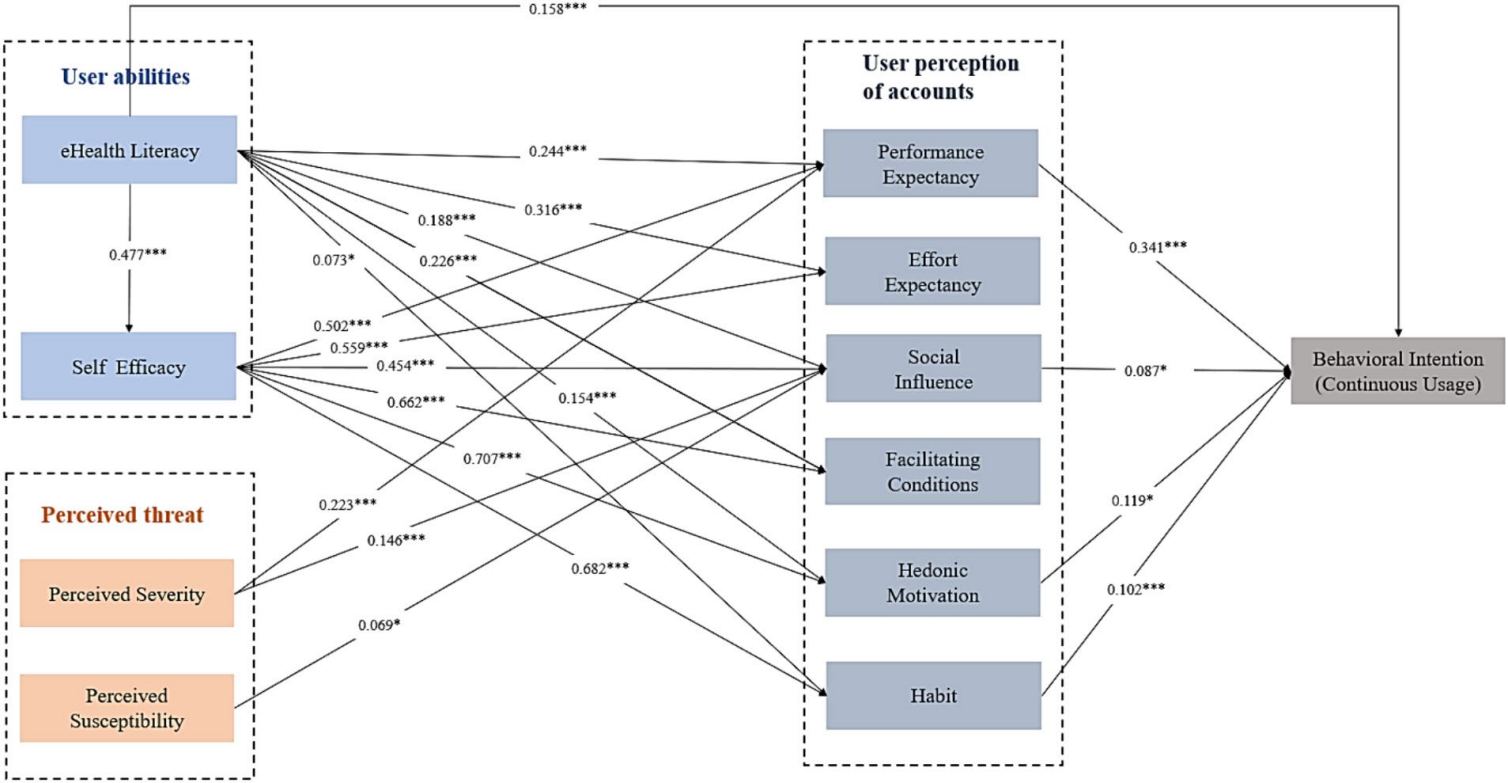
Structural model results of the hypostasis.

The structural equation model for the continued intention to use women’s health public accounts is presented in Figure 2, with good model fit indices: χ^2^/df = 2.794, RMSEA = 0.046, CFI = 0.935, and TLI = 0.929. The model results indicated that performance expectancy (*β* = 0.341), social influence (*β* = 0.087), hedonic motivation (*β* = 0.119), habit (*β* = 0.102), and eHealth literacy (*β* = 0.158) have statistically significant effects on the continued intention to use women’s health public accounts. Additionally, perceived severity, perceived susceptibility, and self-efficacy indirectly influence the continued intention to use women’s health public accounts by affecting various dimensions of UTAUT2.

## 4 Discussion

### 4.1 Research conclusion and significance

This study aimed to investigate the factors influencing women’s continued use of women’s health WeChat public accounts, integrating the UTAUT2 model, HBM model, and eHealth literacy theory to construct a comprehensive model. The research findings indicate: (1) At the perception level of public accounts, performance expectancy, social influence, hedonic motivation, and habit directly and positively influence women’s continued intention to use women’s health WeChat public accounts. (2) In terms of personal abilities, individuals with higher eHealth literacy are more inclined to have a strong intention to continue using public accounts. Additionally, eHealth literacy and self-efficacy can indirectly influence the intention to continue use by affecting performance expectancy, effort expectancy, social influence, facilitating conditions, hedonic motivation, and habit. (3) In the perception of threat, perceived severity and perceived susceptibility can indirectly influence the continued intention to use public accounts by affecting performance expectancy and social influence.

This study extended the theoretical model of mobile health technology usage behavior, providing empirical support for understanding women’s continued intention to use women’s health WeChat public accounts and its influencing factors. It also offers theoretical guidance for improving the quality of WeChat public accounts and user retention on WeChat public accounts.

### 4.2 Impact of the UTAUT2 model on the continued intention to use women’s health WeChat public accounts

Consistent with previous research (19, 49–52), this study demonstrated that the public’s performance expectancy, social influence, hedonic motivation, and habit perception directly and positively influence women’s continued intention to use women’s health WeChat public accounts. This suggests that the UTAUT2 model exhibits consistency in explaining the influencing factors of user’s continued intention to use health WeChat public accounts targeting different audiences and content. Among these, performance expectancy remains the most important predictive factor for mobile health usage behavior (7), indicating that users are more willing to continue using women’s health WeChat public accounts when they perceive them as beneficial to their health (50). Recommendations and usage by family and friends can also enhance user loyalty to health WeChat public accounts (19). Additionally, since the content of health WeChat public accounts often involves professional medical knowledge, individuals may find it challenging to accept and adopt complex professional knowledge due to information gaps in the medical field, thus affecting their continued intention to use these public accounts. If the content of the public account is exciting and enjoyable, allowing users to experience fun, they may have a higher acceptance of the health WeChat public account and be more willing to read its content in the long term (53). The hedonic motivation for acceptance and intention to use a product has also been explained in other mobile health technologies (50–52). This study also indicated that habit can promote the continued intention to use women’s health WeChat public accounts. After regular and repeated use of the product, individuals may develop a usage habit, making them more inclined to use the product continuously in the long term, as seen in similar results in studies on online learning platforms (49), health-related mobile apps (52), and other fields. This study did not observe significant effects of effort expectancy and facilitating conditions on women’s health WeChat public accounts. This may be because users’ behaviors on WeChat public accounts typically involve reading, forwarding, and commenting on articles, which are not perceived as difficult or challenging tasks for individuals who have already obtained information from the public account. However, the ease of understanding health knowledge and the availability of assistance when faced with difficulties in understanding are crucial components of effort expectancy and facilitating conditions. This may affect users’ continued intention to use women’s health WeChat public accounts. Therefore, further exploration of the impact of these two factors on the continued behavior of women’s health WeChat public accounts is needed in a larger population. In the realm of health communication practices, women’s health WeChat public accounts should fully explore women’s specific preferences and needs regarding health, providing more useful health knowledge tailored to women. Additionally, health communicators should clarify the benefits of adopting health information for users, helping them expect that adopting specific health behaviors will be effective. They should also strive to make the content of women’s health WeChat public accounts more concise and clearer, using straightforward and easy-to-understand language to explain women’s health knowledge. This will minimize the barriers for users to comprehend and adopt health information. Meanwhile, women tend to value the enjoyment of searching for health information online more than men do (54). Therefore, public accounts related to women’s health should focus on enhancing both the quality and the interestingness of their educational content to increase women’s continuance intention. Additionally, compared to men, women’s search behaviors for health-related information are driven more by social motivations and are more likely to be influenced by their family, relatives, and friends (54, 55). Hence, women’s health WeChat public accounts need to emphasize word-of-mouth marketing, boosting sharing, and recommendation rates to enhance the positive impact of social influence on women’s sustained use intentions.

### 4.3 The impact of self-efficacy on women’s continued intention to use women’s health WeChat public accounts

The results of this study indicated that self-efficacy has a positive effect on performance expectancy, effort expectancy, social influence, facilitating conditions, hedonic motivation, and habit, thereby indirectly influencing women’s continued intention to use women’s health WeChat public accounts. Self-efficacy is defined as the perceived knowledge and ability of an individual to effectively perform specific tasks using digital devices such as computers (56). Previous research suggested that lower internet self-efficacy is associated with greater difficulty in using mobile technology (57) and a stronger concern about digital health technology (58). On the contrary, individuals with high self-efficacy for health WeChat public accounts perceive a stronger ability to overcome usage obstacles, have more confidence in the positive effects of health WeChat public accounts on their wellbeing, and therefore have higher performance expectancy and effort expectancy (5). They are also more likely to find pleasure in using mobile health technology, promoting the development of positive habitual behaviors. Interestingly, consistent with the hypothesis, self-efficacy also has a positive impact on social influence and facilitating conditions. This may be because individuals who are confident in their ability to effectively use internet technology to obtain and apply health information have more skills and resources. Therefore, they are more likely to accept and continue using mobile health technology under the mobilization of social networks, leading to higher social influence and facilitating conditions. However, in contrast to previous research results (59), this study did not observe a direct effect of self-efficacy on the continued intention to use mobile health technology. Overall, the results of this study expanded on the impact of self-efficacy on technological perception. Compared to men, women tend to perceive themselves as having weaker digital skills (54). Therefore, self-efficacy may have a greater impact on women’s intention to continue using mobile health technologies, highlighting the need for broader mobile technology training and health education to enhance women’s self-efficacy for mobile health technology. Additionally, the user interface and the information provided by women’s health WeChat public accounts should not be overly complex, as this could negatively affect women’s intention to continue using them.

### 4.4 The impact of eHealth literacy on women’s continued intention to use women’s health WeChat public accounts

The results of this study demonstrated that individuals with higher eHealth literacy have a stronger intention to continue using health WeChat public accounts. eHealth literacy is defined as the ability to search, comprehend, and evaluate health information from electronic resources and apply this information to make health decisions (31). eHealth literacy can influence individuals’ health information-seeking behaviors in a new media environment (60, 61) and is one of the core predictors of information technology adoption and continued use behavior (62–64). Previous research indicated that lower levels of eHealth literacy are associated with lower engagement in health information-seeking behaviors (61, 65) and higher levels of technological anxiety (66). On the contrary, individuals with higher eHealth literacy are more adept at obtaining the health information they need through WeChat public accounts, leading to a stronger intention to continue using these accounts. Since eHealth literacy encompasses traditional literacy, information literacy, media literacy, health literacy, scientific literacy, and computer literacy (31), future efforts should focus on enhancing the eHealth literacy of the public through mobile health technology training and health education. This will increase their channels for accessing health information, thereby reducing information gaps and improving health equity.

Furthermore, the study also found that eHealth literacy can indirectly affect women’s continued intention to use health WeChat public accounts by influencing self-efficacy, performance expectancy, effort expectancy, social influence, facilitating conditions, hedonic motivation, and habit. Previous research suggested that higher levels of eHealth literacy are associated with higher levels of self-efficacy (38, 67). According to the knowledge, attitude, and practice (KAP) theory, health knowledge is the foundation for establishing correct beliefs and attitudes (68). Therefore, the higher the ability to access, evaluate, and use health information through electronic media, the higher the self-confidence and efficacy expectations for using online information technology, resulting in higher self-efficacy for online information technology use. Additionally, similar to previous research, individuals with higher eHealth literacy have a higher acceptance level and stronger capabilities for using mobile health technology (69, 70). Therefore, they have positive effects on performance expectancy and effort expectancy and are more likely to derive satisfaction and pleasure from using mobile health technology. For users with lower eHealth literacy, health WeChat public accounts need to enhance the quality, authority, and interest of their content, reduce barriers to understanding professional knowledge, and increase their intention to continue using the public accounts. Interestingly, the study also found that eHealth literacy has positive effects on social influence and facilitating conditions. Since eHealth literacy not only reflects an individual’s cognitive abilities but also serves as a social skill (71), individuals with higher eHealth literacy have more resources for using online health technology and are more likely to continue using mobile health technology under the influence of people around them. The results of this study further emphasize the impact of eHealth literacy on health information behaviors, highlighting the need for strategies such as providing technical education support to improve the eHealth literacy levels of the public in the future.

### 4.5 The impact of perceived health threats on women’s continued willingness to use women’s health WeChat public accounts

This study demonstrated that perceived severity and perceived susceptibility can indirectly affect the sustained usage intention of public accounts by influencing performance expectancy and social influence. In this study, perceived severity refers to the severity of consequences women associate with gynecological diseases, while perceived susceptibility refers to women’s belief in the likelihood of contracting gynecological diseases. Together, they constitute perceived health threats. Previous research indicated that individuals with higher perceived threats are more likely to consider preventive electronic healthcare services useful compared to those in good health (72). A high level of perceived health threats leads to strong health motivation, increasing performance expectations for wearable devices (50). Additionally, higher perceived health threats are associated with stronger intentions for online platform usage (73) and a more positive attitude toward using health applications (74). Therefore, individuals with higher perceived health threats are more likely to be influenced by social environments, resulting in a more frequent acquisition of health information and an increased willingness to use health-related WeChat public accounts. In summary, unlike traditional technologies, research on mobile health technology usage behavior is not only related to technological perception but also to individuals’ cognitive and behavioral aspects related to diseases. Because women tend to be more attentive to potential health threats compared to men (55), women’s health-related WeChat public accounts should enhance health education to increase public attention and awareness of gynecological diseases, thereby fostering sustained usage intentions and promoting healthy behaviors.

### 4.6 Limitations

Although this study has achieved certain results, there are limitations. First, this study is a cross-sectional study. Therefore, it is not possible to determine the causal relationship between variables. Second, the sample of this study is limited to the women attending the obstetrics and gynecology department at a hospital in Hebei Province. Therefore, the results of this study can only reflect the continuous usage of women with or likely to have gynecological diseases in a specific region. The extrapolation of the results of this study is limited because the willingness to consistently use women’s health WeChat public accounts may vary depending on regional economic conditions, cultural contexts, and women’s health conditions. In future studies, expanding the sample range to include more regions and diverse backgrounds of female users could enhance the representativeness of the results. Third, this study used a self-administered questionnaire survey, which may introduce recall bias and subjective evaluation bias regarding the usage behavior and perceptions of WeChat public accounts. Finally, this study explored the continuous use intention of women’s health WeChat public accounts from the perspectives of UTAUT2 and HBM. However, the factors influencing the effectiveness of women’s health communication are diverse. Given women’s specific needs and motivations regarding health communication, it is essential to construct theoretical models tailored specifically to women’s health communication. Therefore, supplementing the questionnaire survey with interviews of typical individuals could provide valuable insights to further refine and enrich the understanding of women’s willingness to sustain the use of women’s health public accounts, offering more effective guidance and support for women’s health information dissemination and user engagement.

## Data availability statement

The raw data supporting the conclusions of this article will be made available by the authors, without undue reservation.

## Ethics statement

The studies involving humans were approved by the Ethics Committee of the Fourth Central Hospital of Baoding City. The studies were conducted in accordance with the local legislation and institutional requirements. The participants provided their written informed consent to participate in this study.

## Author contributions

HM: Formal analysis, Methodology, Software, Visualization, Writing – original draft, Writing – review & editing. JL: Data curation, Investigation, Writing – review & editing. MD: Data curation, Investigation, Writing – review & editing. SH: Data curation, Investigation, Writing – review & editing. XS: Conceptualization, Supervision, Writing – review & editing. TL: Conceptualization, Resources, Writing – review & editing, Data curation, Investigation, Project administration. YW: Conceptualization, Resources, Writing – review & editing, Funding acquisition, Supervision.
